# Neutralizing antibodies targeting a novel epitope on envelope protein exhibited broad protection against flavivirus without risk of disease enhancement

**DOI:** 10.1186/s12929-023-00938-y

**Published:** 2023-06-14

**Authors:** Li-Chen Yen, Hsin-Wei Chen, Chia-Lo Ho, Chang-Chi Lin, Yi-Ling Lin, Qiao-Wen Yang, Kuo-Chou Chiu, Shu-Pei Lien, Ren-Jye Lin, Ching-Len Liao

**Affiliations:** 1 Department of Microbiology and Immunology, National Defense Medical Center, Taipei, Taiwan; 2 National Institute of Infectious Diseases and Vaccinology, National Health Research Institutes, No. 35, Keyan Road, Zhunan, Miaoli County, 35053 Taiwan; 3 Institute of Life Sciences, National Defense Medical Center, Taipei, Taiwan; 4 Institute of Preventive Medicine, National Defense Medical Center, Taipei, Taiwan; 5 Institute of Biomedical Sciences, Academia Sinica, Taipei, Taiwan; 6 Department of Family Dentistry, Tri-Service General Hospital, National Defense Medical Center, Taipei, Taiwan; 7 School of Dentistry, National Defense Medical Center, Taipei, Taiwan; 8 National Mosquito-Borne Diseases Control Research Center, National Health Research Institute, Miaoli, Taiwan

**Keywords:** Japanese encephalitis virus, Dengue virus, Zika virus, Flavivirus vaccine, Neutralizing epitope, Envelop protein domain II

## Abstract

**Background:**

Flavivirus causes many serious public health problems worldwide. However, licensed DENV vaccine has restrictions on its use, and there is currently no approved ZIKV vaccine. Development of a potent and safe flavivirus vaccine is urgently needed. As a previous study revealed the epitope, RCPTQGE, located on the bc loop in the E protein domain II of DENV, in this study, we rationally designed and synthesized a series of peptides based on the sequence of JEV epitope RCPTTGE and DENV/ZIKV epitope RCPTQGE.

**Methods:**

Immune sera were generated by immunization with the peptides which were synthesized by using five copies of RCPTTGE or RCPTQGE and named as JEV-NTE and DV/ZV-NTE_._ Immunogenicity and neutralizing abilities of JEV-NTE or DV/ZV-NTE-immune sera against flavivirus were evaluated by ELISA and neutralization tests, respectively. Protective efficacy in vivo were determined by passive transfer the immune sera into JEV-infected ICR or DENV- and ZIKV-challenged AG129 mice. In vitro and in vivo ADE assays were used to examine whether JEV-NTE or DV/ZV-NTE-immune sera would induce ADE.

**Results:**

Passive immunization with JEV-NTE-immunized sera or DV/ZV-NTE-immunized sera could increase the survival rate or prolong the survival time in JEV-challenged ICR mice and reduce the viremia levels significantly in DENV- or ZIKV-infected AG129 mice. Furthermore, neither JEV -NTE- nor DV/ZV-NTE-immune sera induced antibody-dependent enhancement (ADE) as compared with the control mAb 4G2 both in vitro and in vivo.

**Conclusions:**

We showed for the first time that novel bc loop epitope RCPTQGE located on the amino acids 73 to 79 of DENV/ZIKV E protein could elicit cross-neutralizing antibodies and reduced the viremia level in DENV- and ZIKV-challenged AG129 mice. Our results highlighted that the bc loop epitope could be a promising target for flavivirus vaccine development.

**Supplementary Information:**

The online version contains supplementary material available at 10.1186/s12929-023-00938-y.

## Background

Flaviviruses, single-stranded positive-sense RNA viruses, comprise several medically important viruses, including Dengue virus (DENV), yellow fever virus, Zika virus (ZIKV), Japanese encephalitis virus (JEV), West Nile virus (WNV), and tick-borne encephalitis virus [1, 2]. DENV infection is caused by any of four DENV serotypes (DENV-1, -2, -3, and -4) and can result in illnesses ranging from dengue fever (DF) to severe and life-threatening dengue hemorrhagic fever (DHF) and dengue shock syndrome (DSS) [3]. A ZIKV outbreak was reported in several countries in 2016. ZIKV infection causes severe neurological complications in adults and microcephaly, congenital malformation, and fetal demise in fetuses [4–7].

With global climate change, mosquito-borne disease epidemics appear to be more frequent and diverse. However, despite decades of effort, a live attenuated tetravalent chimeric vaccine, Dengvaxia®, was only recommended in those who have previously had DF, and the overall vaccine efficacy was lower than expected [8]. Recently, there is another dengue vaccine, QDENGA® is a live-attenuated chimeric tetravalent dengue vaccine, which has been developed by Takeda Vaccines Inc. and granted marketing authorization by European Commission (EC) in 2022. The most difference between Dengvaxia® and QDENGA® is that using YFV-17D and DENV-2 PDK-53 as vaccine backbone, respectively. QDENGA® broke the limitation of Dengvaxia®, which can only use in dengue-seropositive persons as the recommend vaccinate strategy, it was proven to be immunogenic and well-tolerated in multiple phase I and II clinical studies, independent of the participants’ age or serostatus [9]. Although QDENGA® stimulated higher levels of neutralizing antibody to DENV-2 but relatively weak protection against DENV-3 and DENV-4 [10–12]. Therefore, it is also required to develop the tetravalent DENV vaccine currently. Efforts to develop a DENV vaccine have included live-attenuated, live-recombinant and inactivated viruses, as well as subunit vaccines based on recombinant proteins and naked DNA constructs [13, 14]. The envelope (E) glycoprotein of DENV had been reported to be responsible for viral attachment through binding to the cellular receptor, and is therefore the most immunologically protein for eliciting the majority of the protective DENV antibody response [15, 16]. The E protein is comprised of three structural domains, designated domain I (DI), DII, and DIII. Previously, a dengue vaccine candidate comprised of consensus E protein domain III (cED III) of DENV was developed, and recombinant cED III immunization in BALB/c mice elicited neutralizing antibodies against four serotypes of DENV [17]. Although the most potent mouse mAbs against flaviviruses bind to DIII [18–20], very few antibodies in humans target this region [21]. Thus, other studies had indicated that cED III-binding antibodies constituted only a small fraction of the total antibodies binding to DENV in human immune sera, and suggested that other regions of the E protein of the virus are primarily responsible for DENV neutralization [22]. In view of this, it is essential to identify critical epitopes of the E protein for the development of safe and efficacious tetravalent DENV vaccines [23].

Furthermore, as the ZIKV outbreak occurred in areas with high DENV exposure, it was observed that anti-DENV antibodies may facilitate ZIKV ADE [24]. Some studies have indicated enhancement of DENV and ZIKV infection in vitro by pan-flavivirus-reactive antibodies [25, 26]. In addition, sera from DENV- and ZIKV-infected patients could also enhance DENV and ZIKV infection in vitro [24, 27, 28]. Besides, passive transfer of cross-reactive antibodies isolated from DENV-, WNV- and ZIKV-infected patients caused more severe illnesses in DENV-infected AG129 or ZIKV-infected *Stat2*^*−/−*^ mice [27, 29]. Thus, any newly developed flavivirus vaccine should protect against DENV and ZIKV without inducing ADE of DENV and ZIKV simultaneously.

Recent studies characterizing 30 DENV cross-neutralizing monoclonal antibodies, and identified that one human mAb, 1C19, could recognize a novel conserved site in E protein domain II of DENV, which was located on the bc loop (amino acids 73 to 79, RCPTQGE) [30]. This 1C19 human antibody could neutralize all four DENV serotypes efficiently, and also possessed the ability to compete for binding against the more common fusion loop antibodies [31], which were reported to target the amino acids 98 to 111 of E protein and contribute to the ADE phenomenon of DENV infection [30]. Therefore, in this study, based on these desirable features, the unique epitope (RCPTQGE) revealed by 1C19 was rationally designed in the development of DENV and ZIKV vaccine.

In this study, we found that DV/ZV-NTE epitope (RCPTQGE) immune sera could cross-neutralize DENV-1/2/3 and ZIKV in vitro. Besides, passive transfer of DV/ZV-NTE and JEV-NTE immune sera in vivo not only reduced the viremia levels in DENV- or ZIKV-infected AG129 mice, DV/ZV-NTE immune sera even prolonged the survival time in JEV-challenged ICR mice. Furthermore, these immune sera did not induce ADE, validated by in vitro and in vivo ADE assays. Our novel findings revealed that the bc loop epitope located on the flavivirus E protein could be applied to develop as a potent and safe flavivirus vaccine.

## Materials and methods

### Cell lines and viruses

BHK-21 cells, the baby hamster kidney cell line (ATCC CCL-10), and C6/36 cells, the mosquito cell line (ATCC CRL-1660), were cultured in RPMI1640 medium containing 5% fetal bovine serum (FBS; Hyclone). Vero cells, the African green monkey kidney cell line (ATCC CCL-81), were grown in MEM containing 10% FBS. K562 cells, a human leukemia cell line (ATCC CCL-243), were maintained in RPMI1640 medium containing 10% FBS. The JEV RP-9 strain and four serotypes of DENV (DENV-1: Hawaii strain, DENV-2: 16681 strain, DENV-3: H87 strain, DENV-4: H241 strain) were propagated in C6/36 cells, and viral titers were determined by focus-forming assays in BHK-21 cells as described previously [32]. The ZIKV PRVABC59 strain was amplified in C6/36 cells, and viral titers were measured by plaque-forming assays in Vero cells, as described previously [33, 34].

### Alignments, peptides synthesis and antibodies generation

Amino acid alignments were performed using DENV1 Hawaii, DENV2 16681, DENV3 H87, DENV4 H241, ZIKV PRVABC59, JEV RP9, WNV 05002688 and YFV AAX47570.1 with MultAlin webserver (http://multalin.toulouse.inra.fr/multalin/). PyMOL-based modeling and alignment of DENV, ZIKV, and JEV E proteins. Protein structures for the E proteins from DENV-1 (PDB: 7A3R), DENV-2 (PDB: 1TG8), DENV-3 (PDB: 1UZG), DENV-4 (PDB: 3UAJ), ZIKV (PDB: 5JHM), and JEV (PDB: 5MV2) were obtained from the Protein Data Bank (PDB). PyMOL was used for modeling and aligning the E protein structures. To create the models, the PyMOL built-in function “fetch” was used to obtain the PDB files of each protein structure. The “align” command was then used to align the E protein structures based on the residues that were selected for comparison (i.e., residues 73–79 in the bc loop region). The exposed bc loop of each virus was annotated with residue numbers using “label” command, and the alignment result of the six viruses was presented by highlighting the conserved bc loops between the viruses.

A series of peptides were synthesized in National Institute of Infectious Disease and Vaccinology (NIIDV) core facility, and peptides were resuspended in DMSO and filtered using a 0.22-μm-pore filter. The primary antibodies were generated at LTK BioLaboratories (Taoyuan, Taiwan), 6 weeks old female BALB/c mice were immune and boosted twice times using Freund’s adjuvants with following peptide: (1) JEV neutralizing epitope-immune sera (JEV-NTE sera): a mouse polyclonal antibody targeting the JEV neutralizing epitope sequence (RCPTTGE RCPTTGERCPTTGERCPTTGERCPTTGE), which was five tandem copies of the JEV epitope sequence RCPTTGE, and (2) DENV/ZIKV neutralizing epitope-immune sera (DV/ZV-NTE sera): a mouse polyclonal antibody targeting the DENV and ZIKV neutralizing epitope sequence (RCPTQGERCPTQGERCPTQGERCPTQGERCPTQGE), which was five tandem copies of the DENV and ZIKV epitope sequence RCPTQGE. The details of the immunizations are as follows. Mice immune subcutaneously (s.c.) with 200 µl containing 30 µg peptide in 100 µl PBS mixed with 100 µl Freund’s complete adjuvant (Sigma). The animals were then boosted twice times by s.c. injection of 100 µl containing 30 µg peptide in 100 µl PBS mixed with 100 µl Freund’s incomplete adjuvant (Sigma) at intervals of 2 weeks. Blood samples were collected by submandibular bleeding 2 weeks after the final dose and centrifuged at 3000 r.p.m. for 5 min. The antibodies were purified by passing through a column coated with indicated peptides. Specific antibody targeting JEV-NTE or DV/ZV-NTE antigen were determined by BCA protein assay (Pierce™ BCA Protein Assay Kit) following the manufacturer’s protocol. Flavivirus group-reactive 4G2 mouse monoclonal antibody (Millipore) directed against the fusion loop of the E protein was used to induce ADE in vitro and in vivo*.*

### Focus reduction neutralization tests (FRNT)

Sera samples were diluted via serial twofold dilutions (starting at 1:8), and then heat-inactivated for 30 min at 56 °C prior to testing. A monolayer of BHK-21 cells in 24-well plates was inoculated with each indicated virus, which had been pre-mixed at 4 °C overnight with pre-immunization or post-immunization sera to a final volume of 0.5 ml. The virus titer prior to pre-mixing was approximately 300 FFU per well. After incubation at 37 °C for 2 h, viral titers were determined using the FFU assay as described previously [32], and the neutralizing antibody titer FRNT_50_ was calculated as the reciprocal of the highest dilution that produced a 50% reduction in FFU as compared with control samples containing the virus alone.

### Mouse model

Outbred female ICR mice were purchased from the BioLASCO Experimental Animal Center (Taipei, Taiwan) and AG129 mice were obtained from National Health Research Institutes (Miaoli, Taiwan). All experiments were performed in the AAALAC-accredited Center of Laboratory Animals at the National Defense Medical Center (Taipei, Taiwan). To study protective immunity against JEV, groups of 4-week-old ICR mice were intraperitoneal (i.p.) immune with JEV-NTE- or DV/ZV-NTE-immune sera and then inoculated with 10^5^ PFU per mouse of JEV and simultaneously with an intracerebral (i.c.) injection of 30 μl PBS on day 1 post immunization to damage the blood brain barrier (BBB), as previously described [35]. The survival rates of the infected mice were monitored daily for 20 days. To determine the protective efficacy against four serotypes of DENV and ZIKV, groups of AG129 mice were i.p. immune with JEV-NTE- or DV/ZV-NTE-immune sera and then challenged with DENV-1 (10^6^ FFU), DENV-2 (10^6^ FFU), DENV-3 (5 × 10^5^ FFU), DENV-4 (5 × 10^6^ FFU) or ZIKV (10 PFU) on day 1 post immunization. Individual mouse sera of each group were collected to detect viral loads by focus-forming assay on day 3 post-infection.

### In vitro ADE assay

Antibody-mediated enhancement of JEV, DENV and ZIKV infectivity was determined by FRNT_50_. mAb 4G2, JEV-NTE- or DV/ZV-NTE-immune sera were heat-inactivated prior to testing and diluted via fourfold serial dilutions (starting at 1:8). Serially diluted sera and each indicated virus were mixed and incubated to form immune complexes for 1 h at 37 °C. K562 cells were mixed with immune complexes (MOI = 0.1) and then incubated for 1.5 h at 37 °C. After washing, the cells were resuspended in fresh medium and incubated for 3 days at 37 °C. Infection protocols with and without virus were performed in parallel as controls. The viral titers of infected cells were determined by focus-forming assay. The fold enhancement was defined as the viral titer of infected cells in the presence of sera divided by the viral titer of infected cells in the absence of sera.

### In vivo ADE assay

AG129 mice were i.p. injected with PBS, 5 µg of mAb 4G2, JEV-NTE- or DV/ZV-NTE-immune sera, then i.p. infected 24 h later with 10^4^ FFU of DENV-4. 80-μl whole-blood samples from each mouse were collected 10:1 (v/v) in acid citrate dextrose solution (1.5 mM citric acid, 8.5 mM sodium citrate, and 13.6 mM dextrose) on day 3 post-infection. Platelets were counted using an automated hematology analyzer (Sysmex KX-21 N Sysmex America, Mundelein, IL, USA). Viremia titers were detected by focus-forming assay. Survival rates were monitored daily for 30 days.

### Statistical analysis

GraphPad Prism 5.0 (GraphPad Software, San Diego, CA) was used for data analysis. The data were analyzed using One-way and Two-way ANOVA for group comparison. Survival curves were analyzed by Log-rank (Mantel-Cox) test. *P* < 0.05 was considered statistically significant.

## Results

### Identification of a potential cross-reactive immunogenic targeting site in flavivirus E protein domain II

**Fig. 1:**
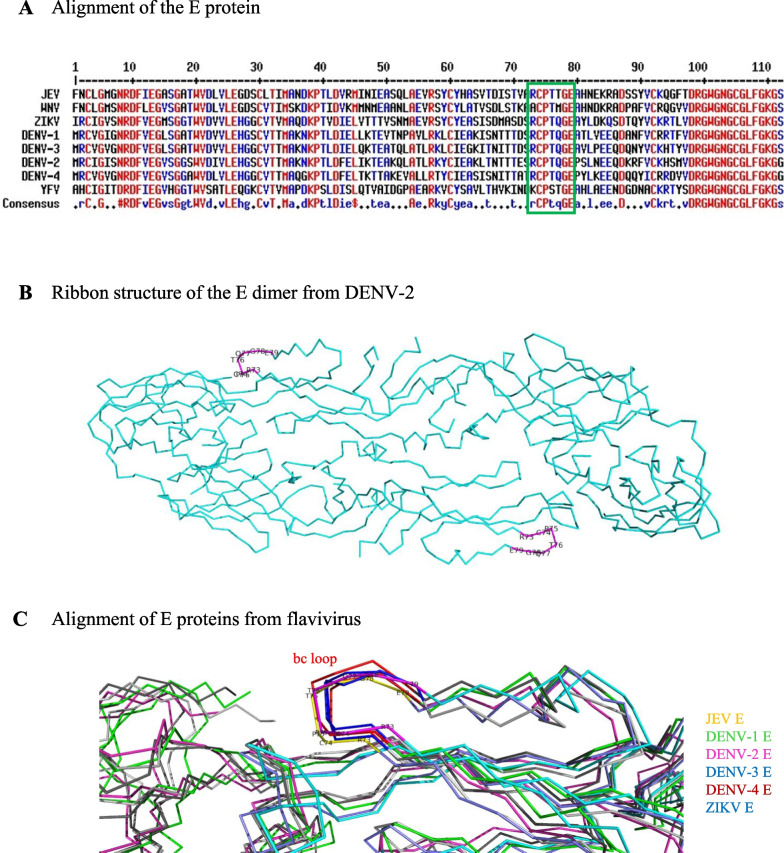
Sequence alignment and PyMOL structural analysis of flavivirus E protein conserved bc loop epitope. **A** Alignment of the E protein amino acid sequences of flaviviruses, highlighting the RCPTTGE and RCPTQGE epitope region with a green box. Amino acid positions are numbered from 1 to 112. Completely conserved epitope residues are labeled in red (aa 74, 75, 78 and 79), while highly conserved epitope residues are colored in blue (aa 73, 76 and 77). **B** Ribbon structure of the E dimer from DENV-2 (PDB: 1TG8) with the bc loop (RCPTQGE) highlighted in magenta and annotated with residue numbers 73–79. **C** Ribbon structures of E proteins from various viruses, including JEV (PDB: 5MV2), DENV-1 (PDB: 7A3R), DENV-2 (PDB: 1TG8), DENV-3 (PDB: 1UZG), DENV-4 (PDB: 3UAJ), and ZIKV (PDB: 5JHM), were aligned using PyMOL. The alignment highlights the conserved bc loops among the viruses, with JEV residues 73–79 (RCPTTGE) and DENV-2 residues 73–79 (RCPTQGE) annotated with residue numbers and colored in yellow and magenta, respectively

In a previous study [30], a bc loop site in the E protein domain II of DENV was targeted by the human mAb clone 1C19. To investigate whether the bc loop sequence in the E proteins of flaviviruses (DENV-1 to DENV-4, ZIKV, and JEV) is conserved, we further aligned the amino acid sequences of these viruses. The results revealed that amino acid positions 73 to 79 (RCPTQGE) in DENV-1 to DENV-4 and ZIKV, and (RCPTTGE) in JEV were aligned together, though with a slight variance in residue 77 for JEV (RCPTTGE) (Fig. 1A). This suggests that this bc loop sequence not only exhibits conservative characteristics but also serves as a potential sequence region for cross-reactive immunogenic targeting sites for these flaviviruses. Furthermore, the structure modeling of DENV-2 (Fig. 1B) and the alignment modeling results of the indicated viruses (Fig. 1C) showed that this sequence in the E dimer proteins was exposed epitopes, providing an explanation for why it can be recognized by antibodies in physiological conditions, as identified in the previous study [36]. Thus, for the subsequent studies of this study, we first synthesized a series of peptides based on the sequence of JEV epitope RCPTTGE and DENV/ZIKV epitope RCPTQGE. The synthesized peptide sequences were repeated for five copies of RCPTTGE or RCPTQGE to be more immunogenic and named based on the results in this study as follows: JEV-neutralizing epitope (JEV-NTE) and DENV/ZIKV-neutralizing epitope (DV/ZV-NTE).

### JEV-NTE- or DV/ZV-NTE-immune sera exhibited immunogenicity and neutralizing capability against flavivirus

**Fig. 2:**
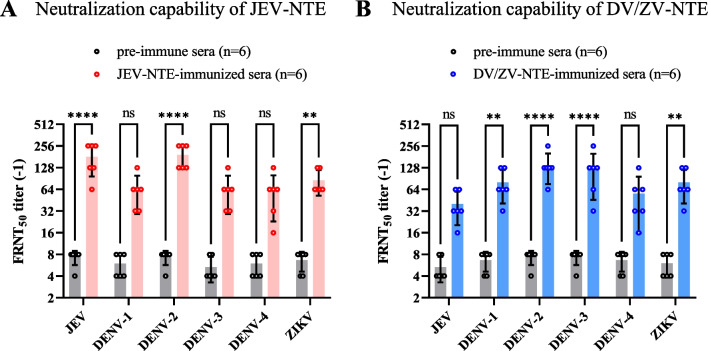
Evaluation of neutralization abilities induced by the synthesized peptide sequences. Anti-sera samples were collected from the mice immune with JEV-NTE or DV/ZV-NTE. The neutralizing titers of **A** JEV-NTE- or **B** DV/ZV-NTE-immune sera were serially diluted from 1:4 to 1:512 with PBS to determine the neutralizing capacities by FRNT_50_. Pre-immune sera (week 0) did not show any neutralization at a 1:8 dilution (the lowest dilution tested), and thus 1:4 was set as the detection limit. Each symbol represents an individual sample, and the horizontal lines indicate the geometric mean values of the groups. One of two representative experiments was shown. Data are the means ± SD of two independent experiments. *P < 0.05; **P < 0.01; ***P < 0.001; ****P < 0.0001 (by Two-way ANOVA). *ns* not significant

To investigate the immunogenicity and neutralization capability induced by the synthesized peptides described above, JEV-NTE- or DV/ZV-NTE-immune sera were generated in the mice and the sera antibody titers were determined by ELISA coated with indicated peptide, respectively. The results showed that both JEV-NTE and DV/ZV-NTE peptide significantly stimulated the production of antibodies (Additional file 1: Fig. S1). Next, focused reduction neutralization tests (FRNT_50_) were performed to assay the neutralizing antibody titers of JEV-NTE- or DV/ZV-NTE-immune sera. The neutralization antibody threshold of > 1:8 was considered positive against flavivirus infection. As shown in Fig. 2, JEV-NTE-immune sera exhibited neutralizing activity towards JEV, DENV-2 and ZIKV, with FRNT_50_ ranging from 64 to 256, significantly higher than those of the control pre-immune sera (Fig. 2A). DV/ZV-NTE-immune sera could neutralize DENV-1/2/3 and ZIKV, with FRNT_50_ titers ranging from 32 to 256, however, the neutralizing activity against JEV and DENV-4 showed no significant difference compared to pre-immune sera, ranging only from 16 to 64 (Fig. 2B). Collectively, we found that DV/ZV-NTE and JEV-NTE-immune sera shows cross neutralizing ability against indicated flavivirus.

### Passive immunization with JEV-NTE- or DV/ZV-NTE-immune sera showed protective abilities in vivo against flavivirus

**Fig. 3:**
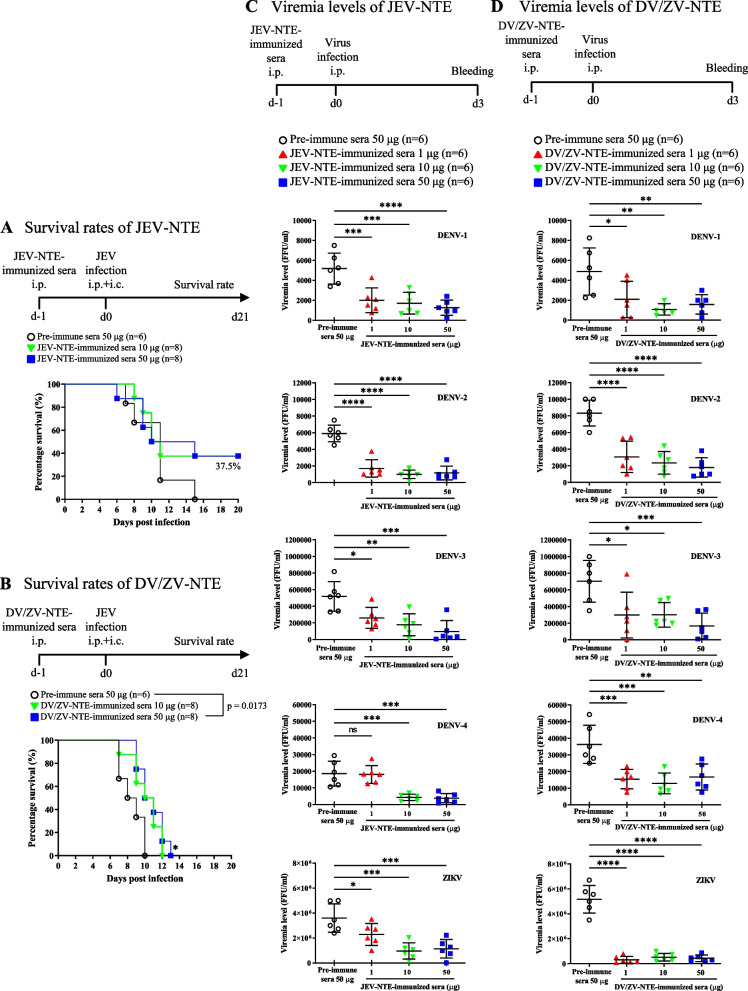
Protective abilities of JEV-NTE- or DV/ZV-NTE-immune sera in vivo. **A**, **B** Groups of 4-week-old ICR mice were first i.p. immune with different doses of **A** JEV-NTE- or **B** DV/ZV-NTE-immune sera, while 50 μg pre-immune sera treatment was used as a control. After 1 day, all the mice were i.p. challenged with 10^5^ PFU of JEV and i.c. injected with 30 μl PBS to break the BBB simultaneously. The numbers of animals (n) in each group are shown. The data are representative results of two independent experiments. The survival rates of the infected mice were monitored daily for 21 days. **C**, **D** Groups of 6-week-old AG129 mice were first i.p. immune with 1, 10 or 50 μg of **C** JEV-NTE- or **D** DV/ZV-NTE-immune sera, and 50 μg pre-immune sera was used as a control. After 1 day, all the mice were i.p. challenged with the indicated four serotypes of DENV and ZIKV. Serum samples were collected from the AG129 mice on day 3 post-infection. Viremia levels of each group were measured from serum samples by focus-forming assay. Each dot represents the viremia level of an individual mouse. Data are the means ± SD of two independent experiments. *P < 0.05; **P < 0.01; ***P < 0.001; ****P < 0.0001 [by Log-rank (Mantel-Cox) test or One-way ANOVA]. *ns* not significant

We next evaluated the protective capabilities of JEV-NTE- or DV/ZV-NTE-immune sera in vivo against flavivirus. First, we examined the protective effect of JEV-NTE- or DV/ZV-NTE-immune sera in outbred ICR mice challenged with JEV via the intraperitoneal (i.p.) plus intracerebral (i.c.) route as previously described [32, 37]. We immunized the ICR mice with each of the indicated anti-sera and then lethal-challenged with JEV as the immunization protocols shown in Fig. 3A, B. Compared with the pre-immune sera, the results showed that passive immunization with 50 μg JEV-NTE-immune sera could increase the survival rate of JEV-challenged ICR mice, although not statistically significant (p = 0.5565). Notably, DV/ZV-NTE-immune sera could significantly prolong the survival rate in the JEV-challenged ICR mice (Fig. 3B). Next, we further investigated the protective efficacy of JEV-NTE- or DV/ZV-NTE-immune sera against four serotypes of DENV and ZIKV infection in AG129 mice, as the immunization protocols shown in Fig. 3C, D. The viremia levels in sera collected from DENV- or ZIKV-infected mice without or with 1, 10 or 50 μg passive immunization of JEV-NTE- or DV/ZV-NTE-immune sera were measured at day 3 post-infection. As compared with the pre-immune control group, immunization with JEV-NTE- or DV/ZV-NTE-immune sera significantly decreased the viremia levels in all serotypes of DENV and ZIKV-infected mice (Fig. 3C, D). Overall, these results demonstrated that passive immunization for both DV/ZV-NTE or JEV-NTE-immune sera could significantly reduce the viremia levels in DENV- and ZIKV- challenged AG129 mice, and DV/ZV-NTE-immune sera could further prolong the survival time in the JEV-challenged ICR mice.

### *JEV-NTE- or DV/ZV-NTE-immune sera reduced the potential risk of ADE *in vitro

**Fig. 4:**
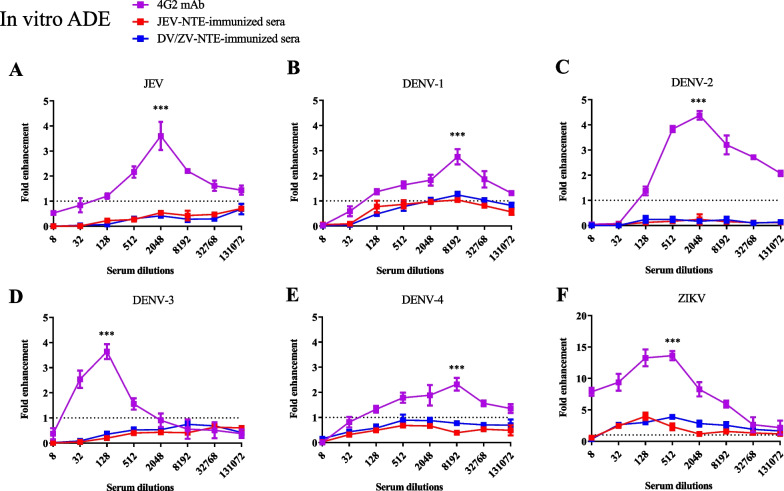
ADE capacities of JEV-NTE- or DV/ZV-NTE-immune sera in vitro. **A**–**F** Comparison of ADE effects in K562 cells by infection (MOI = 0.1) with **A** JEV, **B** DENV-1, **C** DENV-2, **D** DENV-3, **E** DENV-4, or **F** ZIKV in the absence or presence of serial fourfold dilutions of mAb 4G2 (purple symbols), JEV-NTE-immune sera (red symbols), or DV/ZV-NTE-immune sera (blue symbols). Commercially available flavivirus monoclonal antibody mAb 4G2 served as the positive control. The viral titers of infected cells were determined by focus-forming assay. The fold enhancement was defined as the viral titer of infected cells in the presence of sera divided by the viral titer of infected cells in the absence of sera. The data are representative results of two independent experiments. Data are presented as the means ± SD of two independent experiments. *P < 0.05; **P < 0.01; ***P < 0.001 (by Two-way ANOVA)

ADE is a significant concern in the development of DENV and ZIKV vaccines since induction of poorly neutralizing cross-reactive antibodies may prime ADE in a secondary infection with a heterologous serotype. First, to evaluate whether JEV-NTE- or DV/ZV-NTE-immune sera induce ADE in vitro, we infected Fc receptor-bearing K562 cells with JEV, DENV-1, DENV-2, DENV-3, DENV-4 and ZIKV, respectively, and then determined the viral titers in infected cells using a focus-forming assay (Fig. 4A–F). Pan-flavivirus monoclonal antibody (mAb) 4G2 directed against the fusion loop of the E protein served as the ADE positive control [38–40], in which enhancements of JEV, DENV and ZIKV infection were observed (Fig. 4A–F, purple symbols). Our results showed that the fold enhancement values of JEV-NTE-immune sera (Fig. 4A–F, red symbols) and DV/ZV-NTE-immune sera (Fig. 4A–F, blue symbols) were notably lower than the values of mAb 4G2 for JEV, DENV-1, DENV-2, DENV-3, DENV-4 and ZIKV, respectively (Fig. 4A–F), indicating that JEV-NTE- or DV/ZV-NTE-immune sera have little or no capacity for inducing ADE in vitro.

### JEV-NTE- or DV/ZV-NTE-immune sera reduced the potential risk of ADE in vivo

**Fig. 5:**
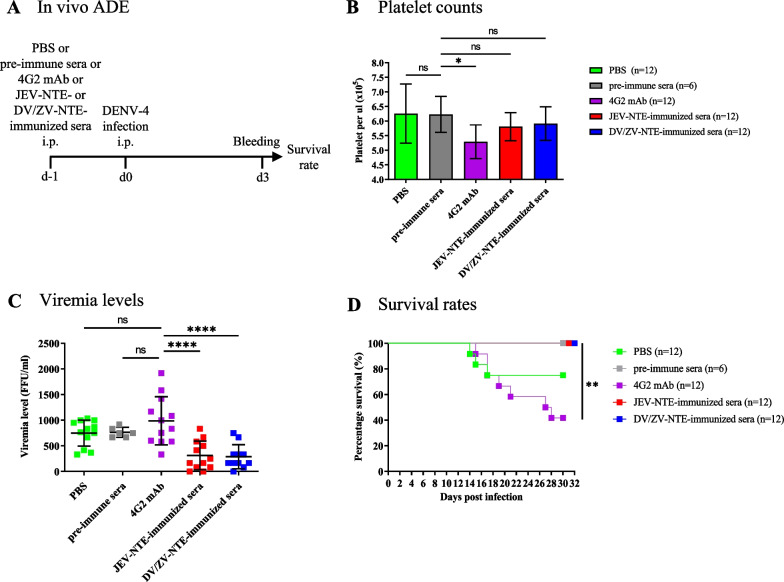
ADE capacities of JEV-NTE- or DV/ZV-NTE-immune sera in vivo. **A** Schematic representation of the in vivo ADE study design. AG129 mice were first i.p. injected with PBS, pre-immune sera, 5 µg of mAb 4G2, JEV-NTE- or DV/ZV-NTE-immune sera, then 24 h later i.p. challenged with 10^4^ FFU of DENV-4. Blood samples were collected at day 3 post-infection and survival rates were monitored for 30 days. **B** Platelets in the blood were counted using an automated hematology analyzer. **C** Viremia levels in the serum were measured by focus-forming assay. Each dot represents the viremia level of an individual mouse. **D** Survival rates were monitored daily for 30 days. The numbers of animals (n) in each group are shown. Statistical differences in survival rates were evaluated by the log-rank test. The data are representative results of two independent experiments. Data are presented as the means ± SD of two independent experiments. *P < 0.05; **P < 0.01; ***P < 0.001; ****P < 0.0001 [by Log-rank (Mantel-Cox) test or One-way ANOVA]. *ns* not significant

Furthermore, while anti-flavivirus antibodies have the potential to cause ADE, mice receiving these antibodies and challenged with sub-lethal doses of flavivirus would develop a more severe form of the disease compared to the negative control group (e.g., PBS or pre-immune sera) [41–44]. To determine the optimal dose of ADE-positive control mAb 4G2, AG129 mice were i.p. injected with PBS or different dose of mAb 4G2, after 24 h, mice were challenged with 10^4^ FFU of DENV-4 (Additional file 1: Fig. S2A) [39, 45]. The results showed that compared to the control group (PBS), AG129 mice receiving 5 µg mAb 4G2 had significantly lower platelet counts, higher viremia and the lowest survival rate (Additional file 1: Fig. S2B–D). Thus, we decided to use 5 µg of mAb 4G2 as the positive control for the subsequent in vivo ADE experiment. Next, we examined whether JEV-NTE- or DV/ZV-NTE-immune sera could induce ADE in vivo (Fig. 5). As shown in the immunization protocol in Fig. 5A, AG129 mice received an i.p. injection of PBS, 5 µg of mAb 4G2 or JEV-NTE- or DV/ZV-NTE-immune sera, followed 24 h later by i.p. challenge with a sub-lethal dose (10^4^ FFU) of DENV-4, respectively. Platelet counts in the blood and viremia levels in the serum collected from the DENV-4-infected mice were detected at day 3 post-infection, and survival rates were monitored for 30 days. The results showed that the platelet counts in the mAb 4G2-injected mice were significantly lower than those in the DENV-4-infected mice, characteristic of the ADE phenomenon. Notably, JEV-NTE- or DV/ZV-NTE-immune sera treated mice exhibited comparable platelet counts to the DENV-4-infected mice (PBS and pre-immune sera group) without statistical significance (Fig. 5B). In addition, the serum viremia levels were lower in the JEV-NTE- or DV/ZV-NTE-immune sera groups than in the mAb 4G2 group (Fig. 5C). Furthermore, administration of JEV-NTE- or DV/ZV-NTE-immune sera in AG129 mice after inoculation with 10^4^ FFU of DENV-4 resulted in 100% survival compared to 40% survival in the mAb 4G2-injected mice (Fig. 5D). Overall, the antibodies elicited by JEV-NTE or DV/ZV-NTE have little capacity for ADE, whereas remarkable ADE was observed in the mAb 4G2-treated mice in vivo.

## Discussion

Flavivirus-associated infections cause serious public health problems worldwide, and there are no widely available vaccines or specific therapeutics for humans. Development of a potent and safe flavivirus vaccine is urgently needed. As the conventional tetravalent DENV vaccine formulated with four individual vaccine components would cause immune dominance of a particular antigenic component of a specific DENV serotype, thus, instead of using four separate components of DENV, development of a single vaccine with representative epitopes or consensus sequences from JEV, four serotypes of DENV, or ZIKV could be a superior vaccine strategy. In this study, we rationally employed the JEV or DENV/ZIKV epitope sequence, RCPTTGE or RCPTQGE (Fig. 1), and further synthesized five copies of these epitope sequence which were named as JEV-NTE and DV/ZV-NTE.

Immunization of the mice with JEV-NTE or DV/ZV-NTE peptide could elicit antibody production (Additional file 1: Fig. S1) and neutralize indicated flavivirus (Fig. 2). Notably, DV/ZV-NTE-immune sera not only exhibited cross-neutralizing ability against DENV-1/2/3 and ZIKV (Fig. 2B), but also significantly reduced the viremia levels on DENV and ZIKV-challenged AG129 mice, and prolonged the survival time of JEV-challenged ICR mice (Fig. 3). However, JEV-NTE- or DV/ZV-NTE-immune sera did not neutralize WNV (data not shown), which may be due to both the 73rd and 77th amino acid sequences of WNV differing from JEV, DENV, and ZIKV (Fig. 1). In summary, the sequence of amino acid residue in the bc loop was crucial for neutralizing different flavivirus members.

A previous study suggested that other regions except for domain III on the E protein of flavivirus are primarily responsible for DENV neutralization [22], and consistent with that, we demonstrated that use of multiple copies of the epitope based on the bc loop sequence of E protein is a potent strategy for DENV/ZIKV vaccine development. Besides, compared to a previous study that focused on the E-dimer-dependent epitopes (EDE) [21], the bc loop was a novel epitope without including in the EDE region. Our findings provided the potential of using the bc loop epitope as a promising cross-virus vaccine candidate and further applied for flavivirus vaccine development.

Antibody-dependent enhancement (ADE) of infection is another important issue in the development of vaccines against DENV and ZIKV. To address this matter, we further examined the capacities of JEV-NTE- or DV/ZV-NTE-immune sera to mediate ADE of JEV, DENV or ZIKV infection by in vitro and in vivo ADE assays. Our results showed that JEV-NTE- or DV/ZV-NTE-immune sera exhibited little or no capacity for ADE of JEV, DENV or ZIKV infection as compared with positive control mAb 4G2 in K562 cells (Fig. 4). Furthermore, JEV-NTE- or DV/ZV-NTE-immune sera elicited normal platelet numbers in blood samples, whereas mAb 4G2 significantly decreased the platelet count, a phenomenon corresponding to severe Dengue infection, such as DHF/DSS (Fig. 5B). In addition, JEV-NTE- or DV/ZV-NTE-immune sera reduced the viremia levels (Fig. 5C) and protected AG129 mice from DENV-4 infection (Fig. 5D) as compared with the mAb 4G2 group, in which enhanced virus infection caused mortality (Fig. 5). Collectively, as it is a serious concern that anti-flavivirus antibodies may enhance JEV, DENV and ZIKV infection, our results showed that JEV-NTE- or DV/ZV-NTE-immune sera could not induce the risk of ADE of JEV, DENV and ZIKV in vitro and in vivo. Further study will be performed to examine active immunization with JEV-NTE or DV/ZV-NTE under different vaccination regimens in mice challenged with JEV, DENV and ZIKV. Taken together, our results showed that a novel bc loop epitope sequence could induce cross-neutralizing ability against indicated flaviviruses without ADE risk. These findings could be used as a basis for future research in the development of pan-flavivirus vaccine.

## Conclusions

We showed for the first time that the novel epitope sequence RCPTQGE that located on the amino acids 73 to 79 of flavivirus E protein could confer cross-neutralization against JEV, DENV and ZIKV. Our results highlighted that the bc loop epitope could be a potential target for flavivirus vaccine development.

## Supplementary Information

**Additional file 1: Figure S1.** Evaluation of the immunogenicities induced by synthesized peptide sequences. Anti-sera were collected from groups of the mice immunized with JEV-NTE or DV/ZV-NTE. The JEV-NTE or DV/ZV-NTE-specific antibody titers were determined by ELISA coating with synthesized peptide RCPTTGE or RCPTQGE, respectively. Pre-immune serumwas used to determine basal levels for comparison. Dotted lines indicated detection limits. Data are shown as the mean ± SD of two independent experiments. **Figure S2.** Determination the optimal dose of ADE-positive control mAb 4G2.Schematic representation of the in vivo ADE study design. AG129 mice were first i.p. injected with PBS, 2.5, 5, 10 or 20 µg of mAb 4G2, then 24 h later i.p. challenged with 10^4^ FFU of DENV-4. Blood samples were collected at day 3 post-infection and survival rates were monitored for 30 days.Platelets in the blood were counted using an automated hematology analyzer.Viremia levels in the serum were measured by focus-forming assay. Each dot represents the viremia level of an individual mouse.Survival rates were monitored daily for 30 days. The numbers of animalsin each group are shown. Statistical differences in survival rates were evaluated by the log-rank test. The data are representative results of two independent experiments. Data are presented as the means ± SD of two independent experiments. *P < 0.05; **P < 0.01 [by Log-ranktest or One-way ANOVA]. ns: not significant.

## Data Availability

The datasets used and analyzed in the current study are available from the corresponding author on reasonable request.
