# Supplementary material for: Neutralizing antibodies targeting a novel epitope on envelope protein exhibited broad protection against flavivirus without risk of disease enhancement
Source: J Biomed Sci. 2023 Jun 14;30:41. doi: 10.1186/s12929-023-00938-y (PMC10265553; doi:10.1186/s12929-023-00938-y)
Supplement: Supplementary file 1 — Additional file 1: Figure S1. Evaluation of the immunogenicities induced by synthesized peptide sequences. Anti-sera were collected from groups of the mice immunized with JEV-NTE or DV/ZV-NTE. The JEV-NTE or DV/ZV-NTE-specific antibody titers were determined by ELISA coating with synthesized peptide RCPTTGE or RCPTQGE, respectively. Pre-immune serumwas used to determine basal levels for comparison. Dotted lines indicated detection limits. Data are shown as the mean ± SD of two independent experiments. Figure S2. Determination the optimal dose of ADE-positive control mAb 4G2.Schematic representation of the in vivo ADE study design. AG129 mice were first i.p. injected with PBS, 2.5, 5, 10 or 20 µg of mAb 4G2, then 24 h later i.p. challenged with 104 FFU of DENV-4. Blood samples were collected at day 3 post-infection and survival rates were monitored for 30 days.Platelets in the blood were counted using an automated hematology analyzer.Viremia levels in the serum were measured by focus-forming assay. Each dot represents the viremia level of an individual mouse.Survival rates were monitored daily for 30 days. The numbers of animalsin each group are shown. Statistical differences in survival rates were evaluated by the log-rank test. The data are representative results of two independent experiments. Data are presented as the means ± SD of two independent experiments. *P < 0.05; **P < 0.01 [by Log-ranktest or One-way ANOVA]. ns: not significant. [file 12929_2023_938_MOESM1_ESM.pptx]

## Slide 1
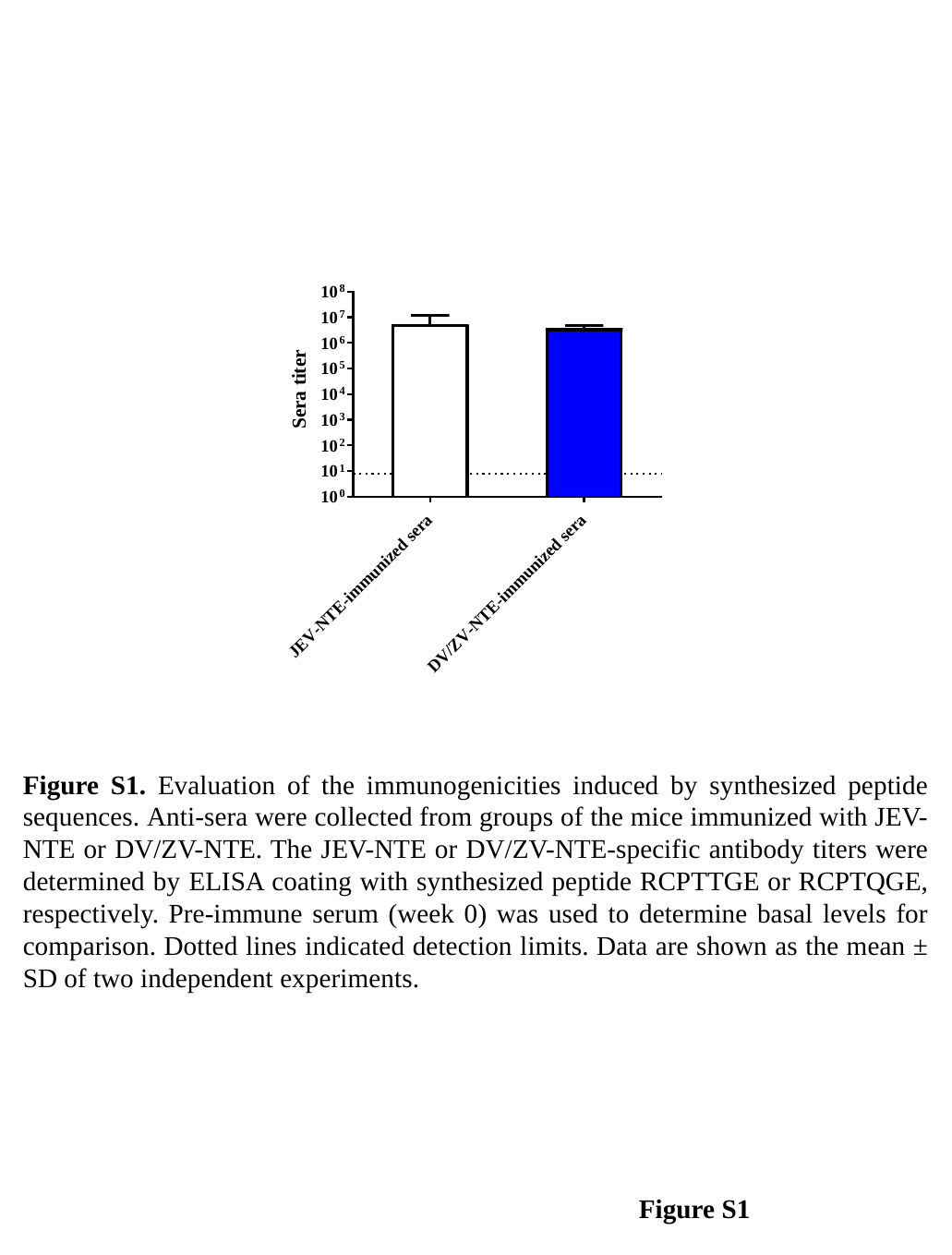

Figure S1. Evaluation of the immunogenicities induced by synthesized peptide sequences. Anti-sera were collected from groups of the mice immunized with JEV-NTE or DV/ZV-NTE. The JEV-NTE or DV/ZV-NTE-specific antibody titers were determined by ELISA coating with synthesized peptide RCPTTGE or RCPTQGE, respectively. Pre-immune serum (week 0) was used to determine basal levels for comparison. Dotted lines indicated detection limits. Data are shown as the mean ± SD of two independent experiments.
Figure S1

## Slide 2
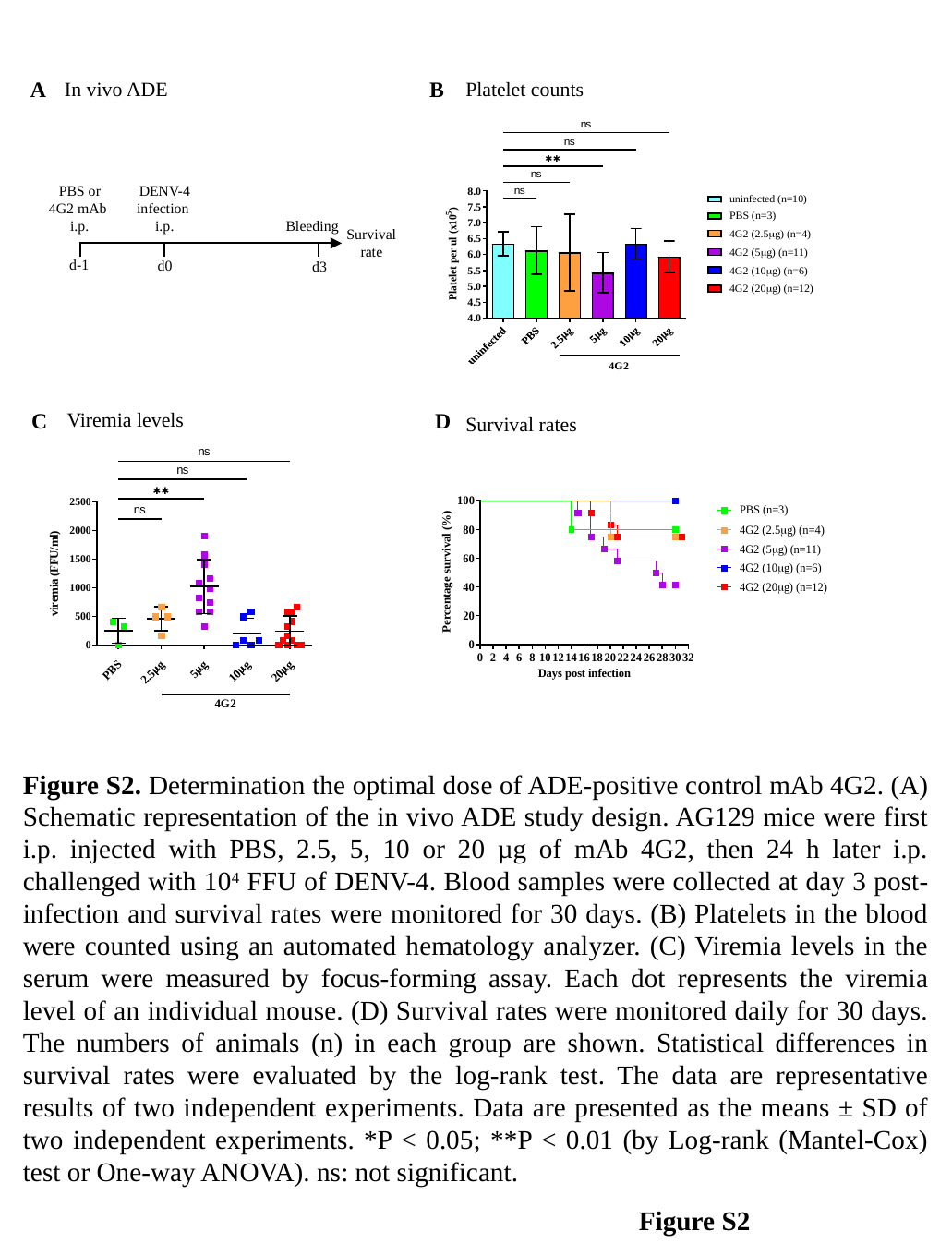

A
B
In vivo ADE
Platelet counts
DENV-4 infection
i.p.
PBS or
4G2 mAb
i.p.
Bleeding
d-1
d0
d3
Survival rate
D
C
Viremia levels
Survival rates
Figure S2. Determination the optimal dose of ADE-positive control mAb 4G2. (A) Schematic representation of the in vivo ADE study design. AG129 mice were first i.p. injected with PBS, 2.5, 5, 10 or 20 µg of mAb 4G2, then 24 h later i.p. challenged with 104 FFU of DENV-4. Blood samples were collected at day 3 post-infection and survival rates were monitored for 30 days. (B) Platelets in the blood were counted using an automated hematology analyzer. (C) Viremia levels in the serum were measured by focus-forming assay. Each dot represents the viremia level of an individual mouse. (D) Survival rates were monitored daily for 30 days. The numbers of animals (n) in each group are shown. Statistical differences in survival rates were evaluated by the log-rank test. The data are representative results of two independent experiments. Data are presented as the means ± SD of two independent experiments. *P < 0.05; **P < 0.01 (by Log-rank (Mantel-Cox) test or One-way ANOVA). ns: not significant.
Figure S2
